# Automated Measurement of Effective Radiation Dose by ^18^F-Fluorodeoxyglucose Positron Emission Tomography/Computed Tomography

**DOI:** 10.3390/tomography10120151

**Published:** 2024-12-23

**Authors:** Yujin Eom, Yong-Jin Park, Sumin Lee, Su-Jin Lee, Young-Sil An, Bok-Nam Park, Joon-Kee Yoon

**Affiliations:** 1 Department of AI Mobility Engineering, Ajou University, Suwon 16499, Republic of Korea; eomyj2@ajou.ac.kr; 2 Department of Nuclear Medicine and Molecular Imaging, Ajou University School of Medicine, Suwon 16499, Republic of Korea; yjpark@aumc.ac.kr (Y.-J.P.); suesj202@aumc.ac.kr (S.-J.L.); aysays@aumc.ac.kr (Y.-S.A.); curies@aumc.ac.kr (B.-N.P.)

**Keywords:** ^18^F-FDG, positron emission tomography, computed tomography, effective dose, deep learning

## Abstract

Background/Objectives: Calculating the radiation dose from CT in ^18^F-PET/CT examinations poses a significant challenge. The objective of this study is to develop a deep learning-based automated program that standardizes the measurement of radiation doses. Methods: The torso CT was segmented into six distinct regions using TotalSegmentator. An automated program was employed to extract the necessary information and calculate the effective dose (ED) of PET/CT. The accuracy of our automated program was verified by comparing the EDs calculated by the program with those determined by a nuclear medicine physician (n = 30). Additionally, we compared the EDs obtained from an older PET/CT scanner with those from a newer PET/CT scanner (n = 42). Results: The CT ED calculated by the automated program was not significantly different from that calculated by the nuclear medicine physician (3.67 ± 0.61 mSv and 3.62 ± 0.60 mSv, respectively, *p* = 0.7623). Similarly, the total ED showed no significant difference between the two calculation methods (8.10 ± 1.40 mSv and 8.05 ± 1.39 mSv, respectively, *p* = 0.8957). A very strong correlation was observed in both the CT ED and total ED between the two measurements (r^2^ = 0.9981 and 0.9996, respectively). The automated program showed excellent repeatability and reproducibility. When comparing the older and newer PET/CT scanners, the PET ED was significantly lower in the newer scanner than in the older scanner (4.39 ± 0.91 mSv and 6.00 ± 1.17 mSv, respectively, *p* < 0.0001). Consequently, the total ED was significantly lower in the newer scanner than in the older scanner (8.22 ± 1.53 mSv and 9.65 ± 1.34 mSv, respectively, *p* < 0.0001). Conclusions: We successfully developed an automated program for calculating the ED of torso ^18^F-PET/CT. By integrating a deep learning model, the program effectively eliminated inter-operator variability.

## 1. Introduction

Medical radiation is the second largest source of radiation exposure, surpassed only by natural background radiation [1]. The repeated use of diagnostic medical imaging procedures that emit ionizing radiation increases the risk of cancers, thereby necessitating proper management [2,3]. In Korea, it has been reported that the estimated population attributable fraction for all cancers due to diagnostic medical procedures is 0.9%, corresponding to 1915 cancer incidences and 637 cancer-related deaths in 2013 [4].

Positron emission tomography (PET) with ^18^F-fluorodeoxyglucose (FDG) is a noninvasive diagnostic tool valuable for the staging, management, and prognostication of various malignant tumors [5]. When combined with computed tomography (CT), which provides an anatomical localization of lesions, hybrid PET/CT offers superior diagnostic accuracy compared to PET alone. Additionally, ^18^F-FDG PET/CT has been utilized for cancer screening in healthy volunteers due to its advantage of whole-body coverage and high specificity [6].

PET/CT is a significant source of medical radiation, with low-dose CT contributing substantially to the overall radiation dose of PET/CT [7,8,9]. The radioactivity of radiopharmaceuticals injected for PET imaging primarily depends on the patient’s weight. In contrast, radiation exposure from CT varies more widely due to multiple factors, including the patient’s weight, tube current, tube voltage, scan range and scan time, ranging from sub-mSv to tens of mSv [10,11,12]. Calculating the radiation dose from PET/CT is complex, time-consuming and burdensome for nuclear medicine physicians, particularly for CT, which requires whole-body image segmentation. The results may vary depending on the method and the operator.

Several CT dosimetry programs are currently available to calculate organ-absorbed doses, effective doses (ED) or both. However, these programs typically use standard human phantoms instead of individualized segmentation data, potentially leading to inaccurate estimates of whole-body CT radiation doses [13]. Furthermore, variations among dosimetry programs arise from differences in the phantoms used [13,14]. Many existing methods rely on separate calculation tools for CT and PET, where radiation doses are assessed independently. Some CT dosimetry programs also require manual data input by users [15,16,17,18], which increases the risk of human error and adds significant time and labor demands. Consequently, hospitals often do not provide information on PET/CT radiation exposure, leaving patients unaware of the amount of radiation they are being exposed to [19,20]. To date, no automated program that can simultaneously calculate radiation doses from both CT and PET has been developed.

Recently, Jacob Wasserthal developed a publicly available CT segmentation program, TotalSegmentator, which leverages deep learning technology [21]. Based on the nnU-Net framework, it is designed to segment 117 anatomical structures, including organs, bones, muscles, and vessels.

This study aimed to develop an automated program that measures radiation doses from ^18^F-FDG PET/CT while minimizing labor and eliminating operator variability. By integrating a deep-learning based segmentation model (TotalSegmentator) into our program, we aimed to streamline the calculation process and enhance the accuracy in radiation dose estimation.

## 2. Materials and Method

### 2.1. FDG PET/CT Protocol

In this study, ^18^F-FDG PET/CT imaging was performed using either the Discovery STE PET/CT scanner or the Discovery MI PET/CT scanner, both of which are products of GE Healthcare (Milwaukee, WI, USA). Prior to the PET/CT examination, all participants were required to fast for a minimum of 6 h. The blood glucose levels of the participants at the time of ^18^F-FDG injection were maintained below 150 mg/dL.

The imaging protocol began with the acquisition of unenhanced CT images, which spanned from the top of the skull to the upper thigh, encompassing the torso. Immediately after the CT scan, emission torso PET data were acquired. The protocol for the Discovery STE PET/CT scanner was as follows: an ^18^F-FDG dosage of 5.1 MBq/kg, a frame duration of 180 s in three-dimensional mode, and a matrix size of 128 × 128 for PET. The CT parameters were 120 kV, a rotation of 17.5 mm, a tube rotation time of 0.5 s/rev, an acquisition time of 58.6 s, and a matrix size of 512 × 512. In contrast, the protocol for the Discovery MI PET/CT scanner was slightly different: an ^18^F-FDG dosage of 4.0 MBq/kg and a frame duration of 90 s in three-dimensional mode for PET. The CT parameters were 120 kV, a rotation of 39.4 mm, and an acquisition time of 50.0 s. CT images from both scanners were acquired using automated tube current modulation mode. The number of CT slices was 263, 299 or 335 slices for the older scanner and 341, 395 or 449 slices for the newer scanner. The PET images were reconstructed using an ordered-subset expectation-maximization algorithm with 20 subsets and two iterations, and the attenuation correction was based on the CT data.

### 2.2. Collection and Preprocessing of PET/CT Data

**Figure 1:**
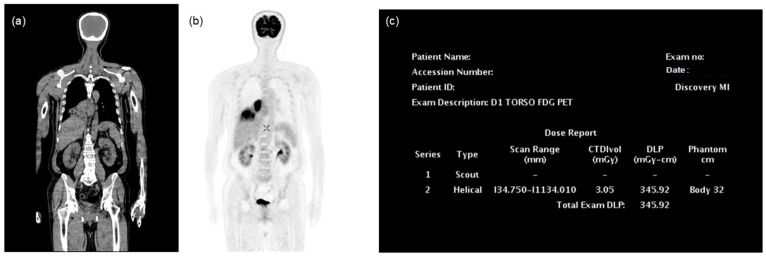
Representative images of PET/CT and a CT dose report from a patient with lung cancer. (**a**) coronal CT (**b**) coronal PET (**c**) CT dose report.

CT and PET images of each patient’s torso were retrieved in the Digital Imaging and Communication in Medicine (DICOM) format from the Picture Archiving and Communication System (PACS, INFINITT Healthcare, Seoul, Republic of Korea) (Figure 1). To calculate the CT ED via an automated program, the CT images were converted from DICOM to Neuroimaging Informatics Technology Initiative (NIfTI) format. Subsequently, the NIfTI files were used as input for the TotalSegmentator (ver. 2.2.1, available at https://github.com/wasserth/TotalSegmentator, accessed on 25 March 2024) [21]. We have incorporated this into our automated program for the segmentation of whole-body CTs. In addition, CT dose reports were collected to acquire necessary information for the calculation of CT ED, including the Volume Computed Tomography Dose Index (CTDIvol) and Dose Length Product (DLP) of the torso (Figure 1). The reliability of the segmentation by TotalSegmentator was visually checked in all CT data.

### 2.3. Effective Dose Calculation

**Figure 2:**
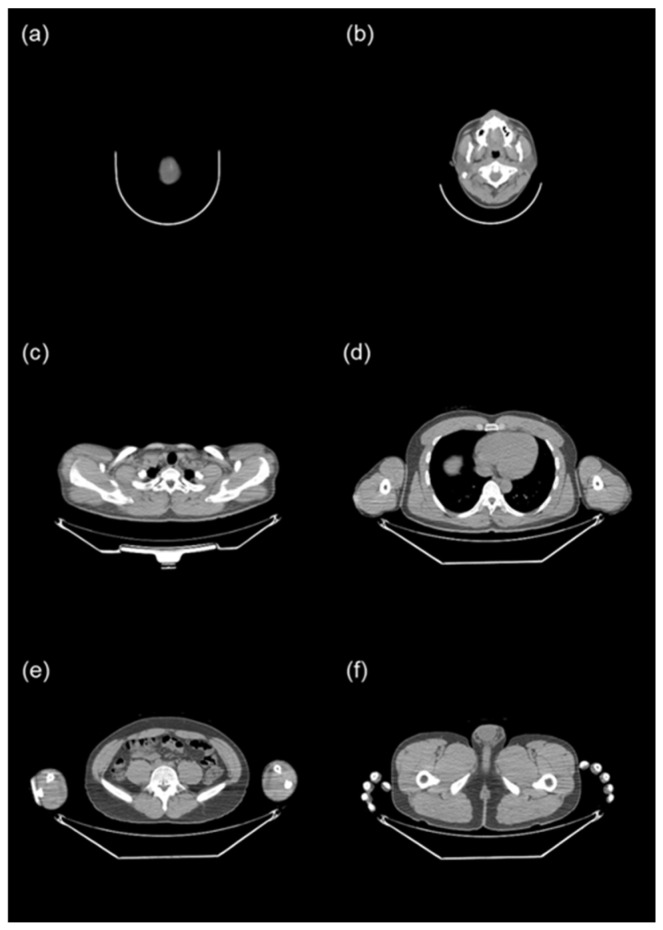
Reference images used for segmenting the torso into six body parts. (**a**) Top of the skull (**b**) C1 (**c**) T1 (**d**) hepatic dome (**e**) iliac crest (**f**) bottom of the pelvic bones.

Given that torso CT encompasses several organs, each with distinct tissue conversion factors, we initially segmented it into six body parts: the head (from the top of the skull to C1), the neck (from C1 to T1), the chest (from T1 to the hepatic dome), the abdomen (from the hepatic dome to the iliac crest), the pelvis (from the iliac crest to the bottom of the pelvic bones), and the femur (from the bottom of the pelvic bones to the last slice) (Figure 2). The top of the skull was identified as the fourth slice above from the first slice of the brain, rather than the first slice of the head, because the TotalSegmentator occasionally recognized the radius as the skull when a PET/CT was performed with the arm raised. The iliac crest was defined as the first slice of the iliac bone.

The CT ED was defined as the sum of the EDs of each body part (1), calculated by multiplying the DLP of each body part by its respective conversion factor (2). The DLP for each body part was determined by multiplying the irradiated length of the body part by CTDIvol (3). The irradiated length was calculated by dividing the torso DLP by torso CTDIvol (4). The CT ED (in mSv) can be calculated using the following formulas:

(1) $$CT\;ED\left(mSv\right)=\sum \text{Part’s}\;ED\left(mSv\right)$$

(2) $$\text{Part’s}\;ED\left(mSv\right)=\text{Part’s}\;DLP\left(mGy\times cm\right)\times Conversion\;factor\left(\frac{mSv}{mGy\times cm}\right)$$

(3) $$\text{Part’s}\;DLP\left(mGy\times cm\right)=\frac{Number\;of\;\text{Part’s}\;slice}{Number\;of\;Total\;slice}\times Irradiated\;length\left(cm\right)\times CTDIvol\left(mGy\right)$$

(4) $$Irradiated\;length\left(cm\right)=\frac{DLP\;(mGy\times cm)}{CTDIvol\;(mGy)}$$

In this study, we opted to use the irradiated length instead of the scan range length. The radiation dose, when calculated using the scan range length, could be underestimated compared to the actual values. This is because the irradiated length tends to be longer than the scan range length when CT images are acquired in a helical rotation [22]. The conversion factors, as adopted from ICRP 102, are as follows: 0.0021 mSv/mGy∙cm for the head, 0.0059 mSv/mGy∙cm for the neck, 0.014 mSv/mGy∙cm for the chest, 0.015 mSv/mGy∙cm for the abdomen, 0.015 mSv/mGy∙cm for the pelvis, and 0.015 mSv/mGy∙cm for the femur [23].

PET ED was defined as the product of the total radionuclide dose and age-specific weighting factor (5). The PET ED (in mSv) can be calculated using the following formula:

(5) $$PET\;ED\left(mSv\right)=Total\;radionuclide\;dose\left(MBq\right)\times Weighting\;factor\;\left(\frac{mSv}{MBq}\right)$$

The weighting factors for ^18^F-FDG, as adopted from ICRP 128, are as follows: 0.095 mSv/MBq for ages 0 to 1 year, 0.056 mSv/MBq for ages 2 to 5 years, 0.037 mSv/MBq for ages 6 to 10 years, 0.024 mSv/MBq for ages 11 to 15 years, and 0.019 mSv/MBq for ages 16 and older [24]. The ED of PET/CT was calculated by adding the EDs of CT and PET scans.

### 2.4. Automated Effective Dose Calculation Program

In this study, we have developed a program that automatically calculates the ED of ^18^F-FDG torso PET/CT scans using a Python script (ver. 3.6). The inputs for the ED calculation program include CT images (in NIfTI format), PET images (in DICOM format), a dose report (in DICOM format), and anatomical part information from TotalSegmentator (in NIfTI format) for each patient. These inputs were uploaded into a single folder. The program is designed to locate the path and extract the necessary information for ED calculation.

**Figure 3:**
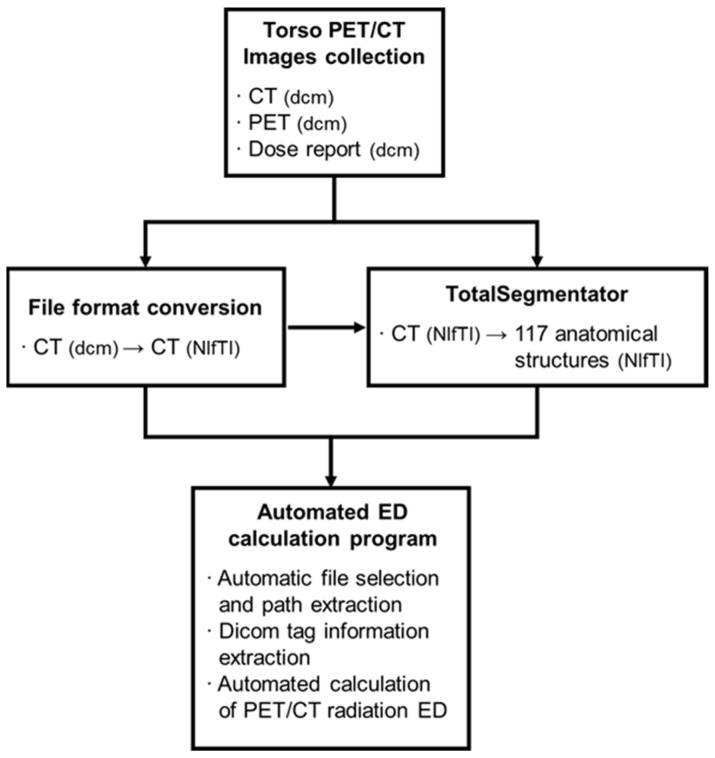
The diagram of the automated ED calculation program for ^18^F-FDG PET/CT.

The program utilized the *z*-axis information from CT images (in NIfTI format) to calculate the total number of slices. It also identified the maximum and minimum values for the brain, C1, T1, liver, and hip and subsequently calculated the lengths of each body part. To determine the range of the abdomen and the pelvis, the right and left hips were unified as a single entity, referred to as the hip. Information on DLP, CTDIvol, the total dose of radionuclide, and age were obtained from the DICOM tags of dose reports and PET images (Figure 3).

### 2.5. Verification of the Automated ED Calculation Program

We prepared two datasets to verify the automated ED calculation program. The first dataset comprises 30 consecutive ^18^F-FDG PET/CT scans performed in March 2024 for preoperative staging of cancers (Group 1). All scans in Group 1 were acquired using a newer scanner model (GE Discovery MI, GE Healthcare, Milwaukee, WI, USA). Using this dataset, we conducted three evaluations: (1) a comparison of the CT ED and total ED calculated by the automated program with those calculated by a nuclear medicine physician (J-K.Y.), the ‘CT-Expo’ program (ver. 2.5, SASCRAD), and whole-body conversion factors (WB CF, 0.0082 and 0.0015) [25,26,27]; (2) an assessment of the correlation between the EDs derived from the automated program and those determined by the nuclear medicine physician; and (3) an evaluation of the program’s repeatability and reproducibility using ED calculations from ten randomly selected PET/CT scans.

The second dataset includes 84 paired ^18^F-FDG PET/CT scans from 42 patients, conducted from April 2023 to April 2024 for either staging or monitoring the therapeutic response of cancers (Group 2). Each pair of PET/CT scans were acquired using different equipment (either older or newer PET/CT scanners) within a one-year period. With this dataset, we evaluated whether our automated program worked properly with both scanners, as the newer scanner incorporates significantly different technologies. In addition, we assessed whether there was a significant difference in radiation dose between these scanners and investigated the causes of this difference.

The clinical characteristics of the patients in Group 1 (n = 30) and Group 2 (n = 42) are shown in Table 1. Group 1 consisted of 12 male and 18 female patients, with an average age of 60 ± 11 years. Group 2 comprised 24 male and 18 female patients, with an average age of 60 ± 12 years. The mean body weight was 60.4 ± 12.5 kg for Group 1 and 59.4 ± 11.3 kg for Group 2. In Group 1, lung cancer was the most common disease, accounting for 8 out of 30 patients (26.7%), followed by endometrial cancer, which affected 7 out of 30 patients (23.3%). In Group 2, lymphoma was the most common disease, accounting for 7 out of 42 patients (16.7%), with lung cancer being the second most common, affecting 6 out of 42 patients (14.3%).

In Group 2, 21 patients underwent ^18^F-FDG PET/CT scans using an older scanner for the initial imaging, followed by a newer scanner for follow-up imaging. The remaining 21 patients underwent the process in reverse order. The mean interval between the initial imaging and follow-up imaging for Group 2 was 165 ± 76 days.

### 2.6. External Validation of the Automated ED Calculation Program

For the external validation of the automated program, we collected 34 ^18^F-FDG PET/CT scans acquired from 25 other hospitals between September and November 2024, which were brought by patients upon transfer (Group 3). All 34 scans were performed to evaluate patients with cancers. Sixteen scans were acquired using PET/CT scanners from Siemens Healthineers (four with Biograph 128, six with Biograph 64, three with Biograph 40, and three with Biograph 16, Forchheim, Germany). Twelve scans were acquired using PET/CT scanners from GE Healthcare (seven with Discovery MI, four with Discovery 710, and one with Discovery 690, Milwaukee, WI, USA). The remaining six scans were acquired using PET/CT scanners from Philips Healthcare (four with Gemini TF and two with Ingenuity TF, Eindhoven, The Netherlands). We aimed to determine whether our program could measure the radiation dose from these scans and to investigate the causes of any measurement failures.

### 2.7. Statistical Analysis

Data are represented as the mean ± standard deviation. The Student’s *t*-test was employed to compare the CT ED, PET ED and total ED between the nuclear medicine physician and the automated program, and between the older and newer PET/CT scanners. A *p*-value of less than 0.05 was considered statistically significant. The correlation coefficient (r^2^) was also determined. Bland–Altman plots were used to display the differences (with a 95% confidence interval) in EDs as calculated by the nuclear medicine physician and the automated program. The maximum allowed differences were calculated as described [28]. Statistical analyses were performed using Medcalc software (ver. 22.030, Medcalc Software Ltd., Ostend, Belgium) or RStudio (AGPL ver. 3). To evaluate the repeatability of the automated program, intraclass correlation coefficients were analyzed.

## 3. Results

### 3.1. Comparison of PET/CT EDs: Automated Program vs. Conventional Methods

**Figure 4:**
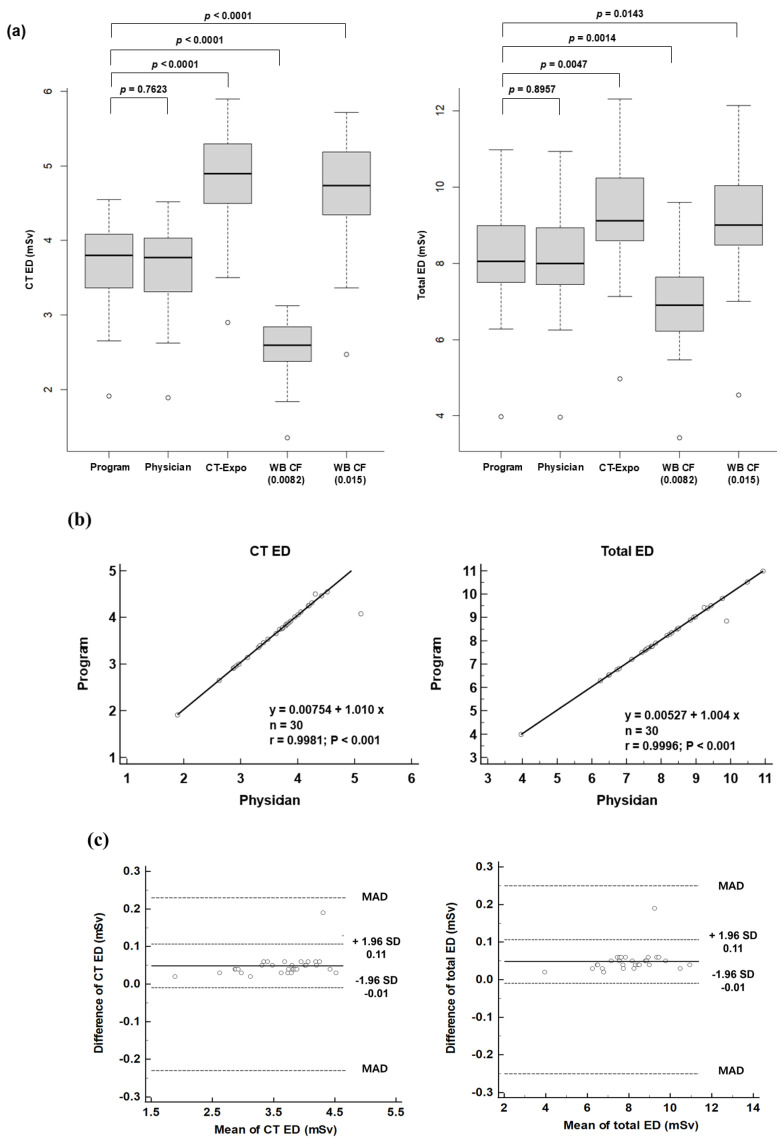
(**a**) Box and whisker plots of effective doses for CT and PET/CT (total) calculated by various methods; automated program, nuclear medicine physician, CT-Expo’ program and whole-body conversion factors (0.0082 and 0.0015). (**b**) Correlation between effective doses calculated by the automated program and the nuclear medicine physician. (**c**) Bland–Altman plots for the differences in effective doses of CT and PET/CT (total) calculated by the automated program and a nuclear medicine physician; MAD, maximum allowed difference.

Table 2 displays the EDs of ^18^F-FDG PET/CT calculated using various methods. Figure 4a compares the EDs for CT and PET/CT as determined by the automated program, a nuclear medicine physician, the ‘CT-Expo’ program, and WB CFs.

The CT ED calculated by the automated program (3.67 ± 0.61 mSv) showed no significant difference compared to that calculated by the nuclear medicine physician (3.62 ± 0.60 mSv, *p* = 0.7623). However, the CT ED from the automated program was significantly lower than those obtained using the ‘CT-Expo’ program (by 30.2% (4.78 ± 0.74 mSv, *p* < 0.0001)) or WB CF (0.015) (by 26.1% (4.63 ± 0.75 mSv, *p* < 0.0001)) and significantly higher than that calculated using WB CF (0.0082) (by 31.1% (2.53 ± 0.41 mSv, *p* < 0.0001)). The PET ED was identical for both the automated program and the nuclear medicine physician (4.43 ± 0.86 mSv), as it depends on the injected dose of ^18^F-FDG and the patient’s age. Similarly, there was no significant difference in total EDs between the automated program and the physician (8.10 ± 1.40 mSv and 8.05 ± 1.39 mSv, respectively, *p* = 0.8957). In contrast, the total ED calculated by the automated program was significantly lower than those obtained using the ‘CT-Expo’ program (by 13.7% (9.21 ± 1.52 mSv, *p* = 0.0047)) or WB CF (0.015) (by 11.8% (9.06 ± 1.53 mSv, *p* = 0.0143)) and significantly higher than that calculated using WB CF (0.0082) (by 14.1% (6.96 ± 1.22 mSv, *p* = 0.0014)).

The CT and total EDs calculated by the automated program demonstrated a very strong correlation with those calculated by the physician (r^2^ = 0.9981 for CT and r^2^ = 0.9996 for PET/CT, Figure 4b). The regression analysis revealed slopes of 1.010 and 1.004, with intercepts of 0.008 and 0.005 for the CT ED and total ED, respectively.

Figure 4c illustrates the differences in EDs between the automated program and the nuclear medicine physician. For CT ED, the difference was 0.05, with a limit of agreement (LoA) ranging from −0.01 to 0.11. Similarly, for the total ED, the difference was 0.05, with a LoA ranging from −0.01 to 0.11. The maximum allowed differences, calculated from the coefficients of variation of both methods, were 0.23 for the CT ED and 0.25 for the total ED. One patient exhibited a higher CT ED (0.19) and total ED (0.19) than the upper LoA, but both values were within the range of the maximum allowed difference. The other values were within the LoAs for both the CT ED and total ED.

To assess repeatability and reproducibility, ED measurements were repeated three times for 10 randomly selected PET/CT scans at a different time point from the initial measurement. The results showed that all repeated ED calculations were identical across the 10 scans and consistent with the initial measurements (Supplementary File S1). Interclass correlation coefficients for the CT ED, PET ED and total ED were 1.0, 1.0 and 1.0, respectively. These findings demonstrate that the automated program exhibits excellent repeatability and reproducibility in measuring PET/CT ED.

### 3.2. Comparison of PET/CT EDs: Older Scanner vs. Newer Scanner

The average tube currents of CT scanners were 61.1 ± 9.8 mA (ranging from 35.4 to 77.0 mA) for the newer scanner and 66.3 ± 0.7 mA (ranging from 64.1 to 68.2 mA) for the older scanner. The scan lengths were 1099 ± 35 mm for the newer scanner and 1052 ± 42 mm for the older scanner. The injected doses of ^18^F-FDG were 228.3 ± 45.1 MBq for the newer scanner and 318.6 ± 59.2 MBq for the older scanner.

**Figure 5:**
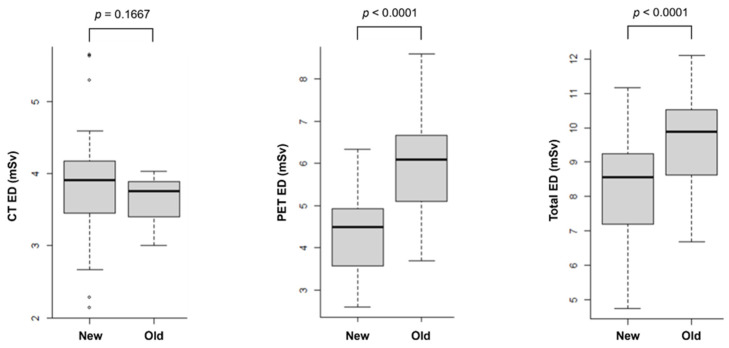
Box and whisker plots of effective doses for CT, PET, and PET/CT (total) from newer and older scanners.

Figure 5 shows the EDs of CT, PET and PET/CT calculated from both newer and older scanners. The ED for CT was similar between the newer and older scanners (3.65 ± 0.26 mSv and 3.83 ± 0.75 mSv, respectively, *p* = 0.1667). Using 1 mSv as a cutoff, the CT ED was higher with the newer scanner in four patients (5.30 vs. 2.93 mSv; 5.63 vs. 3.89 mSv; 4.50 vs. 3.33 mSv; and 5.65 vs. 3.92 mSv). In contrast, the CT ED was higher with the older scanner in three patients (2.14 vs. 3.37 mSv; 2.67 vs. 3.89 mSv; and 2.28 vs. 3.40 mSv).

Meanwhile, the PET ED was significantly lower with the newer scanner (4.39 ± 0.91 mSv) compared to the older scanner (6.00 ± 1.17 mSv, *p* < 0.0001). Consequently, the total ED for PET/CT was also significantly lower with the newer scanner (8.22 ± 1.53 mSv) than with the older scanner (9.65 ± 1.34 mSv, *p* < 0.0001).

For the 21 patients who underwent PET/CT examinations using the newer scanner followed by the older scanner, the average weight change was −2.4 ± 4.2 kg. The CT ED did not differ significantly between the two examinations (3.96 ± 0.70 mSv for the newer scanner vs. 3.70 ± 0.27 mSv for the older scanner, *p* = 0.141). However, the PET ED was significantly lower with the newer scanner (4.61 ± 0.95 mSv) compared to the older scanner (6.26 ± 1.29 mSv, *p* < 0.001). Consequently, the total ED was significantly reduced with the newer scanner (8.58 ± 1.46 mSv) compared to the older scanner (9.96 ± 1.44 mSv, *p* < 0.001).

In a separate group of 21 patients who initially underwent PET/CT examinations using the older scanner and subsequently the newer scanners, the average weight change was −0.4 ± 3.1 kg. Consistent with the first group, there was no significant difference in the CT ED between the two scanners (3.60 ± 0.25 mSv for the older scanner vs. 3.69 ± 0.79 mSv for the newer scanner, *p* = 0.638). Similarly, the PET ED showed a significant reduction with the newer scanner (4.18 ± 0.84 mSv) compared to the older scanner (5.73 ± 0.99 mSv, *p* < 0.001). Consequently, the total ED was significantly lower with the newer scanner (7.87 ± 1.56 mSv) compared to the older scanner (9.34 ± 1.17 mSv, *p* < 0.001).

### 3.3. External Validation of the Automated ED Calculation Program

The automated program successfully calculated EDs for 18 out of 34 scans (52.9%). For these scans, the EDs were 3.67 ± 1.89 mSv (range: 1.83–10.31) for CT, 4.85 ± 1.12 mSv (range: 3.10–6.68) for PET, and 8.52 ± 2.47 mSv (range: 5.99–16.99) for PET/CT. Measurement failures occurred in 16 scans (47.1%) due to the absence of a dose report form in 12 cases (75%) and the lack of ^18^F-FDG dose information in the DICOM headers in four cases (25%). These issues may have arisen during the process of archiving data from PACS for transfer.

## 4. Discussion

In this study, we successfully developed a program to automate the calculation of the effective radiation dose for torso ^18^F-FDG PET/CT. While several studies have addressed the calculation of EDs for PET/CT, to the best of our knowledge, this is the first study to automate calculation of ED by using a deep learning model. Our results showed that the EDs of the PET/CT obtained by the automated program showed a strong correlation with those calculated by a nuclear medicine physician. Additionally, the CT segmentation in this program was standardized using TotalSegmentator-derived anatomic information, thereby eliminating the inter-operator variability.

PET/CT is a hybrid imaging modality that combines both PET and CT, both of which involve the emission of ionizing radiation. Consistent with our findings, the ED from ^18^F-FDG PET generally remains below 10 mSv per examination [29,30]. Advancements in technology have further reduced radiation exposure by enhancing PET sensitivity without compromising image quality [31]. In contrast, the CT ED varies significantly, with low-dose protocols typically ranging from 1 to 10 mSv, while diagnostic protocols often generally exceed 10 mSv [29,30]. As a result, the total ED can surpass 20 mSv, potentially leading clinicians or patients to reconsider undergoing PET/CT examinations. Unlike PET, the CT component of PET/CT lacks such strict standardization, making the calculation of radiation exposure from torso or whole-body CT scans more complex and challenging.

Radiation exposure from PET/CT can be calculated in various ways. As demonstrated in our study, one common approach involves segmenting the torso into five to six regions and calculating the ED using CTDIvol and DLP values measured by CT scanners in conjunction with tissue conversion factors (DLP method) [32]. However, the DLP method has notable limitations. Its accuracy depends on the size of the phantom used, often leading to an underestimation of the ED, particularly in pediatric patients [33]. Beyond the use of phantoms, how DLP values are determined by CT scanners remains largely unknown. Moreover, the DLP method is less ideal than organ-absorbed dose calculations, as it does not assess radiation risks to individual organs [13]. Despite these drawbacks, the DLP method remains widely used due to its simplicity and practicality. It provides a single, standardized metric, making it convenient for cancer risk assessment related to radiation exposure. Additionally, it is valuable for comparing radiation doses across imaging modalities that emit ionizing radiation, thereby supporting protocol optimization [33,34].

Radiation exposure can alternatively be estimated by inputting CT parameters into dosimetry programs, which calculate both organ-specific and total radiation doses [13]. These dosimetry programs are broadly categorized into three types: stand-alone programs that operate with (e.g., CT-Expo, ImPACT) [8,16,17] or without Microsoft Excel (e.g., ImpactDose, NCICT, WinDose) [35]; PACS-integrated tools (e.g., DoseWatch, Radimetrics) [36]; and web-based platforms (e.g., VirtualDose). Summing the organ-absorbed doses calculated by these tools aligns well with the ICRP definition of ED. Furthermore, these programs allow users to select patient-specific phantoms such as age, gender, and body size [13]. However, limitations persist, particularly regarding the phantoms used in these programs [13]. Variability in computational human phantoms among programs leads to significant differences in ED estimates [14,37,38]. For organs within the scan range, discrepancies as high as 67% have been reported. Additionally, these tools are constrained by limited scanner model options and mismatches between computational human phantoms and actual patient anatomy, which can result in inaccurate dose estimations [13]. Using individualized anatomical models derived from patients instead of standard computational phantoms remains a labor-intensive process [33].

In this study, we compared EDs calculated by an automated program to those obtained using the “CT-Expo” software. As reported in the results, the automated program yielded significantly lower CT and total EDs compared to “CT-Expo”, by 30.2% and 13.7%, respectively. These findings align with previous studies indicating that EDs calculated using the DLP method were generally lower—ranging from 4% to 74%—compared to dosimetry programs, except for head scans [22].

The simplest method to estimate the ED of whole-body CT is to multiply the DLP by whole-body tissue conversion factor [25,26,27]. In this study, applying a conversion factor of 0.0082 mSv/mGy∙cm to Group 1 resulted in estimated CT ED and total ED values that were 31.1% and 14.1% lower, respectively, than those calculated by the automated program. Conversely, using a conversion factor of 0.015 mSv/mGy∙cm produced estimated CT ED and total ED values that were 26.1% and 11.8% higher, respectively, than those calculated by the automated program [39]. Therefore, using a whole-body tissue conversion factor to estimate CT ED can lead to either underestimation or overestimation of the ED for ^18^F-FDG PET/CT.

When comparing older and newer PET/CT scanners, the CT ED was not significantly different. The newer CT scanner had a longer scan length and a higher number of the CT slices, while the average tube current was lower than that of the older scanner. In four patients, the CT ED was more than 1 mSv higher with the newer scanner compared to the older scanner, resulting from the higher tube voltage (140 keV vs. 120 keV) [10]. PET/CT technicians increased the tube voltage to accommodate the body shape of these patients. The tube currents for these CT scans were similar between the two scanners. Conversely, in three patients, the CT ED was lower with the newer scanner compared to the older scanner. In these cases, reduced tube currents (35.4, 38.1 and 44.5 mA) contributed to the lower CT ED, as the device automatically adjusted the tube current settings.

The PET ED was significantly lower in the newer scanner compared to the older scanner. The newer PET scanner has higher sensitivity for gamma photons, leading to a significant reduction in the injected dose of ^18^F-FDG [31]. Additionally, weight changes due to surgery or anti-cancer chemotherapy between the initial and follow-up imaging influenced the difference in PET ED. For the 21 patients who underwent PET/CT scans in the order of older to newer scanners, the PET ED decreased by 3.8% due to a 2.3 kg reduction in weight. In contrast, for the remaining 21 patients who underwent scans in the reverse order, the PET ED decreased by 0.8% due to a 0.4 kg reduction in weight. However, the influence of weight change on the PET ED was relatively small compared to the change in the injected dose. A comparison of PET/CT EDs between an older scanner and a newer scanner confirmed that the automated program functions accurately, regardless of the scanner model and acquisition-reconstruction parameters.

One limitation of this automated program is that when PET/CT is performed with the arms raised, TotalSegmentator may incorrectly identify certain anatomical structures. For example, it may mistakenly recognize the radius as the skull and the carpal bones as the C1 vertebra. Consequently, when dividing the torso into body parts, subtle discrepancies can arise between the actual CT ED and the calculated CT ED due to the incorrect segmentation of the head and neck regions. To address this issue, this study set the top of the skull at four slices above the brain. However, attention must be paid to patients’ arm positions, and the segmentation standards should be adjusted in accordance with the protocols of each institution.

Another limitation is that this study was conducted at a single center. To address this, the automated program was tested on 34 PET/CT scans from other hospitals. We found that the measurement failures were primarily due to the absence of dose report forms and missing dose information in the DICOM files. While these findings provide valuable insights, future multi-center studies are necessary to improve the program’s generalizability and robustness.

## 5. Conclusions

In summary, we developed an automated program to measure radiation doses for ^18^F-FDG PET/CT, demonstrating strong correlation with manually calculated ED. The program exhibited excellent repeatability and reproducibility while effectively reducing inter-operator variability through the integration of a deep learning-based program. Furthermore, it performed reliably regardless of the scanner model or acquisition-reconstruction parameters. By simplifying the workflow to require only file conversion and placement in a designated folder, this automated program is expected to significantly reduce a physician’s workload.

## Figures and Tables

**Table 1:** Clinical characteristics of patients.

		Number of Patients (%)	Mean ± SD
Group 1 (n = 30)			
Age (years)			60 ± 11
Sex	Male/female	12:18 (40.0:60.0)	
Weight (kg)			60.4 ± 12.5
Type of Cancers	Lung	8 (26.7)	
	Endometrial	7 (23.3)	
	Esophageal	3 (10.0)	
	Ovary	3 (10.0)	
	Renal cell	2 (6.7)	
	Breast	1 (3.3)	
	Cervix	1 (3.3)	
	Colon	1 (3.3)	
	Lymphoma	1 (3.3)	
	Nasal	1 (3.3)	
	Rectal	1 (3.3)	
	Vocal cord	1 (3.3)	
Group 2 (n = 42)			
Age (years)			60 ± 12
Sex	Male/female	24:18 (57.1:42.9)	
Weight (kg)			59.4 ± 11.3
Type of Cancers	Lymphoma	7 (16.7)	
	Lung	6 (14.3)	
	Stomach	5 (11.9)	
	Breast	4 (9.5)	
	Rectal	3 (7.1)	
	Renal cell	3 (7.1)	
	Cervix	2 (4.8)	
	Colon	2 (4.8)	
	Pancreatic	2 (4.8)	
	Prostate	2 (4.8)	
	AoV	1 (2.4)	
	Esophageal	1 (2.4)	
	GB	1 (2.4)	
	Head and Neck	1 (2.4)	
	Klatskin	1 (2.4)	
	Ovary	1 (2.4)	

Abbreviations: SD, standard deviation; AoV, Ampulla of Vater; GB, gallbladder.

**Table 2:** Comparison of the CT EDs and total EDs among various methods.

Patient	PET ED	Automated Program		Physician		CT-Expo		WB CF (0.0082 *)		WB CF (0.015 *)	
		CT	Total	CT	Total	CT	Total	CT	Total	CT	Total
1	6.52	4.46	10.98	4.42	10.94	5.80	12.32	3.08	9.60	5.63	12.15
2	5.76	4.06	9.82	4.01	9.77	5.30	11.06	2.84	8.60	5.19	10.95
3	5.96	4.55	10.51	4.52	10.48	5.90	11.86	3.12	9.08	5.72	11.68
4	5.38	4.12	9.50	4.06	9.44	5.30	10.68	2.81	8.19	5.14	10.52
5	5.14	3.89	9.03	3.85	8.99	5.10	10.24	2.68	7.82	4.91	10.05
6	5.15	4.25	9.40	4.19	9.34	5.60	10.75	2.94	8.09	5.38	10.53
7	4.93	4.50	9.43	4.31	9.24	5.60	10.53	2.99	7.92	5.47	10.40
8	4.69	4.31	9.00	4.25	8.94	5.60	10.29	2.95	7.64	5.39	10.08
9	4.46	3.82	8.28	3.79	8.25	4.90	9.36	2.59	7.05	4.74	9.20
10	4.64	4.25	8.89	4.20	8.84	5.60	10.24	2.96	7.60	5.41	10.05
11	4.57	3.78	8.35	3.74	8.31	4.90	9.47	2.59	7.16	4.73	9.30
12	4.77	4.08	8.85	4.03	8.80	5.40	10.17	2.85	7.62	5.22	9.99
13	4.58	3.92	8.50	3.88	8.46	5.10	9.68	2.69	7.27	4.92	9.50
14	4.68	3.86	8.54	3.82	8.50	5.00	9.68	2.68	7.36	4.90	9.58
15	4.16	3.74	7.90	3.68	7.84	4.80	8.96	2.57	6.73	4.70	8.86
16	4.37	3.85	8.22	3.80	8.17	4.90	9.27	2.62	6.99	4.79	9.16
17	4.23	3.40	7.63	3.34	7.57	4.50	8.73	2.39	6.62	4.37	8.60
18	4.23	3.46	7.69	3.40	7.63	4.50	8.73	2.40	6.63	4.40	8.63
19	3.49	4.01	7.50	3.95	7.44	5.10	8.59	2.73	6.22	4.99	8.48
20	4.11	3.65	7.76	3.62	7.73	4.70	8.81	2.50	6.61	4.58	8.69
21	3.67	3.53	7.20	3.48	7.15	4.60	8.27	2.44	6.11	4.47	8.14
22	4.01	3.76	7.77	3.73	7.74	4.90	8.91	2.59	6.60	4.73	8.74
23	4.23	3.36	7.59	3.31	7.54	4.50	8.73	2.38	6.61	4.35	8.58
24	3.67	3.14	6.81	3.12	6.79	4.10	7.77	2.17	5.84	3.97	7.64
25	3.76	3.00	6.76	2.97	6.73	3.90	7.66	2.08	5.84	3.80	7.56
26	3.63	2.65	6.28	2.62	6.25	3.50	7.13	1.84	5.47	3.37	7.00
27	4.8	2.96	7.76	2.92	7.72	3.80	8.60	2.03	6.83	3.71	8.51
28	3.62	2.92	6.54	2.88	6.50	3.80	7.42	2.00	5.62	3.66	7.28
29	3.62	2.91	6.53	2.87	6.49	3.80	7.42	2.02	5.64	3.70	7.32
30	2.07	1.91	3.98	1.89	3.96	2.90	4.97	1.35	3.42	2.47	4.54

Abbreviations: PET, positron emission tomography; CT, computed tomography; ED, effective dose; WB CF, whole-body conversion factor (* mSv/mGy·cm).

## Data Availability

A part of the data is available in a Supplementary File. The rest of the data is available upon reasonable request, by contacting the corresponding author.

## Supplementary Materials

The following supporting information can be downloaded at: https://www.mdpi.com/article/10.3390/tomography10120151/s1, File S1: Repeated measurement of the CT, PET and total EDs using the automated program.
