# Supplementary material for: Automated Measurement of Effective Radiation Dose by 18F-Fluorodeoxyglucose Positron Emission Tomography/Computed Tomography
Source: Tomography. 2024 Dec 23;10(12):151. doi: 10.3390/tomography10120151 (PMC11679132; doi:10.3390/tomography10120151)
Supplement: Supplementary file 1 [file tomography-10-00151-s001.zip › tomography-3384765-supplementary.pdf]

Suppl. File S1. Repeated measurement of the CT, PET and total EDs using the automated program.

Patient	1			2			3		
	**CT**	**PET**	**Total**	**CT**	**PET**	**Total**	**CT**	**PET**	**Total**
2	4.06	5.76	9.82	4.06	5.76	9.82	4.06	5.76	9.82
9	3.82	4.46	8.29	3.82	4.46	8.29	3.82	4.46	8.29
10	4.25	4.64	8.89	4.25	4.64	8.89	4.25	4.64	8.89
13	3.92	4.58	8.50	3.92	4.58	8.50	3.92	4.58	8.50
14	3.86	4.68	8.54	3.86	4.68	8.54	3.86	4.68	8.54
20	3.65	4.11	7.76	3.65	4.11	7.76	3.65	4.11	7.76
22	3.76	4.01	7.77	3.76	4.01	7.77	3.76	4.01	7.77
24	3.14	3.67	6.81	3.14	3.67	6.81	3.14	3.67	6.81
25	3.00	3.76	6.76	3.00	3.76	6.76	3.00	3.76	6.76
26	2.65	3.63	6.28	2.65	3.63	6.28	2.65	3.63	6.28

Abbreviations: PET, positron emission tomography; CT, computed tomography; ED, effective dose.
